# *Bartonella vinsonii* subsp. *berkhoffii* genotype III in a dog with recurrent fever and panniculitis coinfected with *Babesia odocoilei*-like sp.

**DOI:** 10.1093/jvimsj/aalag174

**Published:** 2026-08-24

**Authors:** Alexandra Daravigka, Nektarios Soubasis, Panagiotis Ygeionomakis, Mathios E Mylonakis, Manolis N Saridomichelakis, Rania Farmaki, Alexandros O Konstantinidis, Alexander Civello, Panagiotis Bourdekas, Dimitra Pardali, Emily Kingston, Ricardo G Maggi, Edward B Breitschwerdt

**Affiliations:** Companion Animal Clinic, School of Veterinary Medicine, Faculty of Health Sciences, Aristotle University of Thessaloniki, Thessaloniki 54627, Greece; Companion Animal Clinic, School of Veterinary Medicine, Faculty of Health Sciences, Aristotle University of Thessaloniki, Thessaloniki 54627, Greece; Companion Animal Clinic, School of Veterinary Medicine, Faculty of Health Sciences, Aristotle University of Thessaloniki, Thessaloniki 54627, Greece; Companion Animal Clinic, School of Veterinary Medicine, Faculty of Health Sciences, Aristotle University of Thessaloniki, Thessaloniki 54627, Greece; Clinic of Medicine, Faculty of Veterinary Medicine, University of Thessaly, Trikalon Str. 224, Karditsa GR-43132, Greece; Diagnostic Laboratory, School of Veterinary Medicine, Faculty of Health Sciences, Aristotle University of Thessaloniki, Thessaloniki 54627, Greece; Companion Animal Clinic, School of Veterinary Medicine, Faculty of Health Sciences, Aristotle University of Thessaloniki, Thessaloniki 54627, Greece; Veterinary Pathology Group Histology, Bristol BS7 0BJ, United Kingdom; Private Practitioner, Thessaloniki, Greece; Diagnostic Laboratory, School of Veterinary Medicine, Faculty of Health Sciences, Aristotle University of Thessaloniki, Thessaloniki 54627, Greece; Intracellular Pathogens Research Laboratory, College of Veterinary Medicine, North Carolina State University, Raleigh, NC 27607, United States; Intracellular Pathogens Research Laboratory, College of Veterinary Medicine, North Carolina State University, Raleigh, NC 27607, United States; Intracellular Pathogens Research Laboratory, College of Veterinary Medicine, North Carolina State University, Raleigh, NC 27607, United States

**Keywords:** babesiosis, bartonellosis, canine, coinfections, infectious diseases

## Abstract

A 2-year-old, spayed female, mixed breed dog presented for evaluation of recurrent febrile episodes over 5 months, intermittently accompanied by the development of SC nodules. Histopathologic examination of the SC lesions was indicative of panniculitis. *Bartonella vinsonii* subsp. *berkhoffii* genotype III DNA was amplified and sequenced from an excised SC nodule. Indirect immunofluorescence antibody titer was 1:64, whereas *Bartonella* blood PCR was negative. *Babesia odocoilei*-like species DNA was amplified and sequenced from 2 blood samples obtained 77 days apart at the time of febrile episodes. Treatment with doxycycline, enrofloxacin, and azithromycin for 6 months resulted in a favorable clinical outcome. Fifteen months later, the dog remained healthy. This case indicates that relapsing fever and panniculitis are possible manifestations of *B vinsonii* subsp. *berkhoffii* genotype III infection in dogs. To the best of the authors’ knowledge, infection with a *B odocoilei*-like species has not been reported previously in dogs from Greece.

## Introduction

*Bartonella* spp. are fastidious, facultative intracellular, gram-negative bacteria with worldwide distribution that infect dogs, cats, other animals, and humans.^1^^,^^2^ Transmission to dogs likely occurs via biting arthropods, resulting in persistent intraerythrocytic bacteremia.^2^^,^^3^ A broad clinical spectrum has been associated with bartonellosis in dogs, including fever, endocarditis, myocarditis, pyogranulomatous inflammation, immune-mediated disorders, lymphadenopathy, splenomegaly, cutaneous vasculitis, nodular skin lesions, and panniculitis.^1^^,^^2^^,^^4–9^ Dogs are more commonly infected with *Bartonella henselae* or *Bartonella vinsonii* subsp. *berkhoffii* (*Bvb*) and compared to cats, they are less frequently reservoirs for zoonotic infections.^2^^,^^6^^,^^10^

*Babesia* spp. are intraerythrocytic protozoan parasites, primarily transmitted by ticks. Among more than 100 recognized species, 7 have been identified as pathogens in dogs through DNA sequencing, and cause lethargy, fever, splenomegaly, hemolytic anemia, and thrombocytopenia,^11^ whereas in humans 8 species have been reported.^12^ In both species, severe hemolytic anemia most often is reported in splenectomized or immunocompromised individuals.^12^ Although tick bites are presumably the primary transmission route for all *Babesia* spp., direct transmission between dogs and humans cannot be excluded.^12^^,^^13^  *Bartonella* spp. and *Babesia* spp. coinfections have been documented in both dogs and humans.^13–15^

We describe the clinical presentation, diagnostic evaluation, and treatment outcome for a dog with recurrent febrile episodes and panniculitis that was coinfected with *Bvb* genotype III and a *Babesia odocoilei*-like species.

## Case history

**Figure 1:**
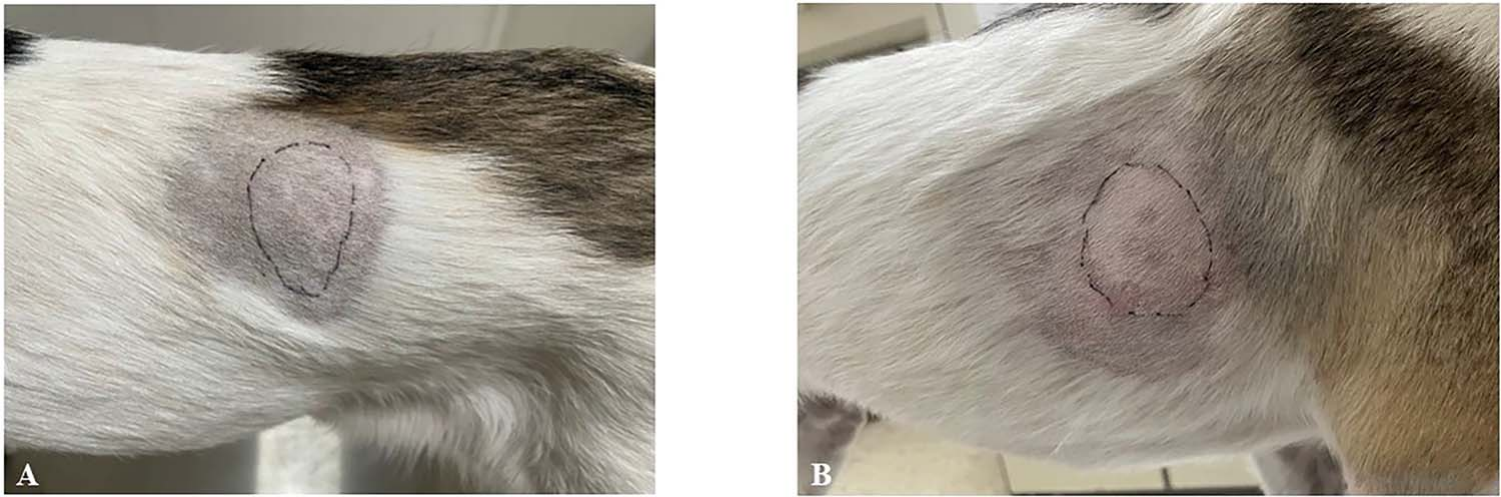
(A) Subcutaneous non-ulcerative, soft, nodular lesions (1.5 × 1.5 cm), located bilaterally on the caudal dorsal trunk of a dog. The black circle outlines the area where the nodules were palpated. (B) Subcutaneous soft nodule (3 × 3 cm), located on the right lateral thorax of a dog. Hair was clipped for lesion visualization. All images were obtained on February 10, 2024.

A 2-year-old, spayed female mixed breed dog residing in the town of Edessa, northern Greece, was referred to the Clinic of Companion Animal Medicine, Aristotle University of Thessaloniki (CAC-AUTh), Greece, for evaluation of recurrent fever. The dog had been rescued from a rural environment, was fully vaccinated, received regular antiparasitic preventive medications, and had lived indoors with a cat since the age of 3 months. A stray cat had been adopted 12 weeks before referral. Five weeks before referral (September 2023), the dog was examined by a general practitioner because of lethargy and fever. On reexamination within 2 days of receiving doxycycline (10 mg/kg PO q24h for 3 weeks) the dog was afebrile. A CBC performed by the referring veterinarian was normal. Serum biochemical analysis documented increased alkaline phosphatase (ALP) activity (473 U/L; reference range 20-150 U/L; see Supplementary Material). The dog was seronegative for *Ehrlichia canis, Borrelia burgdorferi, Anaplasma* spp., and *Dirofilaria immitis* (SNAP 4Dx Plus test, IDEXX). When fever recurred 17 days after doxycycline discontinuation (November 2023), the dog was referred to the CAC-AUTh. Clinical examination, including ocular and orthopedic evaluation, was performed. Pain during joint palpation or lameness was not observed. The dog was febrile (40.2°C), lethargic, and had 2 non-ulcerated, soft, SC nodules (1.5 × 1.5 cm), located bilaterally on the caudal dorsal trunk (Figure 1A). Mild splenomegaly was noted. Findings from the CBC (ADVIA 2120), blood smear examination, serum biochemistry, blood buffy coat cytology, and urinalysis were unremarkable. A repeated SNAP 4Dx Plus test (IDEXX) 5 weeks after the first testing and indirect immunofluorescence assay (IFA) for *Leishmania infantum* were negative. Cytologic examination of fine-needle aspirates (FNAs) from the nodular skin lesions showed the presence of erythrocytes (hemodilution). Thoracic and abdominal radiographs, as well as an echocardiographic examination, evaluated by a radiologist and a cardiologist, respectively, were unremarkable. Abdominal ultrasonography identified a moderate enlargement of the mesenteric, portal, and medial iliac lymph nodes and diffuse splenomegaly. Ultrasound-guided FNA cytology of the mesenteric and medial iliac lymph nodes indicated mild pyogranulomatous lymphadenitis, whereas splenic aspiration cytology results were normal. Joint fluid cytology was unremarkable. Blood, urine, and synovial fluid cultures were negative. The results of the above-mentioned tests are provided in the Supplementary Material. Fever resolved spontaneously within 24 h, and the nodules regressed within 7 days without treatment. Further diagnostic evaluation with cytologic examination of the nodules was recommended but because of the rapid decrease in size, the owner declined the procedure. Follow-up examination was scheduled in 1 month.

One month later (December 2023), fever (40.6°C) recurred with no relapse of skin lesions. Neutropenia (1.1 × 10^9^/L; reference range, 3.9-8 × 10^9^/L; confirmed by blood smear evaluation) and increased ALP activity (712 U/L) were detected. Repeated abdominal ultrasonography was unremarkable, synovial fluid analysis was normal and blood culture was negative. Further investigation included quantitative real-time PCR (qPCR) testing of whole blood (IDEXX Laboratories; Germany) for *Babesia* spp. and *Bartonella* spp., which was negative. Detailed results of the above investigations are available in the Supplementary Material. Fever and neutropenia resolved (neutrophil count: 7.8 × 10^9^/L) spontaneously within 24 h without treatment. At this visit, the owner reported that after adoption of the stray cat, a 10-year-old child in the household developed lymphadenitis, suspected to be caused by *Bartonella* infection, which responded to azithromycin. Because of the suspicion of an undetected *Bartonella* infection in the dog, empirical treatment with doxycycline (10 mg/kg PO q12h, Ronaxan; Boehringer Ingelheim Animal Health, Duluth, GA) and enrofloxacin (10 mg/kg PO q24h, Baytril; Elanco Animal Health, Greenfield, IN) was prescribed for 5 weeks. Appropriate ectoparasite preventive measures were discussed with the owner for both the dog and the cats.

Five weeks after the initiation of antibiotic treatment (January 12, 2024), a fourth febrile episode (>40.0°C) occurred along with the reappearance of the 2 SC nodules at the same location as before, along with a third, larger (3 × 3 cm) SC nodule on the right thoracic wall (Figure 1B). Cytology of all SC lesions indicated sterile pyogranulomatous inflammation, consistent with a diagnosis of panniculitis (see Supplementary Material). Fever resolved within 48 h, and combined antibacterial treatment was continued.

**Figure 2:**
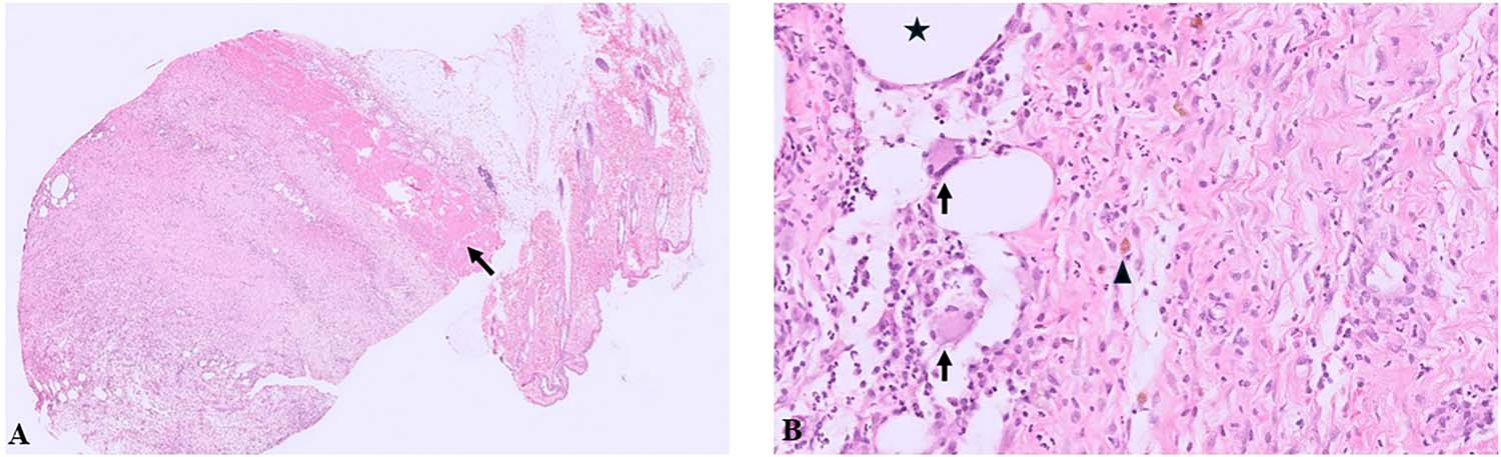
Histology of an incisional biopsy obtained from an SC nodule on the right thoracic wall. (A) Inflammation and fibrosis are centered on the subcutaneous fat deep to the panniculus muscle (arrow). 10 × magnification. (B) Pyogranulomatous inflammation involves neutrophils, macrophages, and occasional multinucleated giant cells (arrows) centered on clear spaces (star) consistent with accumulation of free lipid resulting from loss of adipocytes. Fibrosis is observed to the right of the image. Occasional hemosiderophages (arrowhead) are present indicating concurrent minor chronic hemorrhage. 400 × magnification.

A fifth febrile episode (>40.0°C) occurred 10 days later (January 22, 2024), again resolving within 24 h. A CBC, blood smear examination, and biochemical profile were repeated and the only abnormality was increased ALP activity (671 U/L). The dog was weakly *B henselae* seroreactive (IFA titer, 1:40; Laboklin, Germany; cut-off value < 1:20). An incisional biopsy of the right thoracic wall nodule was obtained aseptically. Histopathology confirmed pyogranulomatous inflammation of adipose tissue without extension to the overlying dermis, consistent with panniculitis. The infiltrate was predominantly neutrophilic, with fewer macrophages and occasional multinucleated giant cells, with accompanying edema, chronic hemorrhage, reactive fibroplasia, and fibrosis (Figure 2). Warthin–Starry silver staining failed to identify organisms. Additional histochemical stains included periodic acid-Schiff, which did not identify fungi, and Ziehl–Neelsen, which did not identify mycobacteria. Real-time qPCR testing of the biopsy specimen was positive for *Bartonella* spp. (IDEXX Laboratories Germany). Findings from these investigations are available in the Supplementary Material. The SC nodules resolved within 1 week. Azithromycin (10 mg/kg PO q24h, Zithromax; Pfizer Inc., New York, NY) was added to the doxycycline and enrofloxacin treatment regimen, and the entire treatment was continued for 6 months. Eighteen days later (February 2024), while on triple antibiotic treatment, a sixth febrile episode (40.2°C) was recorded, lasting 24 h. Increased ALP activity (529 U/L) was the only abnormality (see Supplementary Material).

Because of the relapsing febrile episodes, archived (stored at −20°C) EDTA-anticoagulated blood and serum samples from the dog (obtained during febrile episodes) and from the 2 in-contact cats, along with a cutaneous lesion biopsy specimen from the dog were further examined at the Intracellular Pathogens Research Laboratory, College of Veterinary Medicine, North Carolina State University (IPRL-NCSU). *Bartonella vinsonii* subsp. *berkhoffii* genotype III was amplified and sequenced (392/394 base pair [bp] similarity with *Bvb* genotype III Gen Bank Accession AF143446) from the DNA extracted from the panniculitis lesion (not placed in formalin), using conventional PCR and 425 antisense (as)–1100 sense (s) primers targeting the 16S–23S intergenic transcribed spacer (ITS) region, as previously described.^16^ Of the 6 *Bartonella* species/genotypes tested by IFA, the dog was initially only seroreactive to *Bvb* genotype III (IFA titer 1:64). When tested for evidence of *Babesia, Bartonella*, and *Borrelia* DNA using recently validated digital PCR (dPCR) assays,^12^ the dog was *Piroplasma* 18S rRNA positive for 1 of the 3 blood samples and 3 of 9 enrichment blood culture DNA extractions. In conjunction with ongoing development and validation of *Babesia* ITS1 and ITS2 PCR assays,^17^ concurrent infection with a *Babesia* species, most closely aligning with *B odocoilei* (ITS1, 104/107 bp, 97.2% DNA sequence similarity to GenBank *B odocoilei*  AY339754) was confirmed in 2 blood samples collected 77 days apart*.* In February 2024, the original family cat was *Bartonella* spp. seronegative in all 5 IFA assays and *Bartonella* dPCR positive only in the 21-day enrichment blood culture. The adopted cat was IFA seroreactive to several *Bartonella* species (*Bvb* I: 1:128, *Bvb* II: 1:64, *Bvb* III: 1:64, *B henselae*: 1:256, *B quintana*: 1:128, and *B koehlerae*: < 1:32) but *Bartonella* qPCR and dPCR in blood and enrichment blood cultures were negative. Both cats were *Piroplasma* qPCR and dPCR negative. In June 2024, after 5 months of triple antibiotic treatment, the dog was seroreactive (IFA titer 1:64) for both *Bvb* genotype III and *B henselae* but was qPCR and dPCR negative for *Bartonella* and *Babesia*, respectively.

### Follow-up

After the sixth febrile episode 18 days after the initiation of the triple antibiotic regimen, no additional illness episodes occurred. Findings of CBC and serum biochemistry (different laboratory) remained within reference intervals at 4 and 6 months, including ALP activity (46 and 37 U/L, respectively; reference range, 10-70 U/L). No treatment was administered for *Babesia* spp. infection. Fifteen months after antibiotic discontinuation, no recurrence of panniculitis or fever has been reported, evidenced by the dog’s sustained normal demeanor and rectal temperature measurements performed weekly by the owner.

## Discussion

This dog developed multiple self-limiting or antibiotic-responsive febrile episodes during 5 months, with 3 episodes accompanied by panniculitis. Molecular testing confirmed coinfection with *Bvb* genotype III (SC tissue) and a *Babesia* species (*B odocoilei*-like in blood and enrichment blood culture) that historically infects ruminants and humans. Although the amplification of *Bvb* genotype III DNA from the SC lesion and the response to prolonged antibiotic treatment imply the latter as the primary cause of fever and panniculitis, a potential causative role of the *B odocoilei*-like organism in the febrile episodes cannot be excluded.

Bartonellosis initially was suspected, based on compatible findings (fever, panniculitis, splenomegaly, granulomatous lymphadenitis, neutropenia, and increased ALP activity), exclusion of common endemic vector-borne diseases, and history of lymphadenitis in a family member after the adoption of a stray cat, a known reservoir of *B henselae* and other *Bartonella* species.^1–5^^,^^8^^,^^18^ Previously, *Bvb* genotype III infection has been documented rarely in dogs from Mediterranean countries,^10^^,^^19^ whereas panniculitis in dogs has been associated with *B henselae*,^8^  *Bvb*,^5^ and an undetermined *Bartonella* species.^4^ In our case, *Bvb* was identified in the SC inflamed tissue, similar to the reported case of *B henselae*,^8^ highlighting an association between *Bartonella* infection and panniculitis. Repeated blood cultures were negative for *Bartonella* spp., likely because of intermittent or low-level bacteremia, long-term blood storage and shipment to the IPRL-NCSU, and the fastidious growth requirements of *Bartonella* species.^2^ Compared with qPCR, dPCR can have higher diagnostic sensitivity.^12^^,^^15^ However, blood and enrichment blood culture qPCR and dPCR results were negative, despite amplification and sequencing of *Bvb* genotype III from the skin biopsy specimen. Tissue samples may offer greater sensitivity than blood, because studies have reported higher *Bartonella* DNA prevalence in skin and SC tissue of dogs, suggesting tissue tropism.^1^^,^^20^ Limitations of blood PCR testing were highlighted in a child with seizures in whom enrichment blood culture, qPCR and dPCR were negative, whereas enrichment brain biopsy cultures were PCR positive for *B henselae* DNA.^21^

At different time points, low titers of 1:40 for *B henselae* (commercial laboratory) and 1:64 for *Bvb* genotype III (research laboratory) were documented.^1^^,^^3^  *Bartonella* serology and PCR or enrichment culture-based PCR results often are poorly correlated.^2^ Also, seroreactivity does not confirm causation, because healthy dogs may be seroreactive.^2^ Histopathology and Warthin–Starry silver staining failed to identify organisms, consistent with reports of limited sensitivity of histopathology in visualizing these bacteria.^8^ For unclear reasons, poor correlation exists between the severity of histologic lesions and bacterial numbers in dogs with granulomatous lesions. Despite triple antibiotic treatment, this dog experienced 2 further febrile episodes and 1 episode of panniculitis, raising suspicion for delayed or suboptimal treatment efficacy and supporting the need for prolonged treatment.^2^^,^^14^^,^^22^^,^^23^

In this case, initial diagnostic testing for babesiosis was justified because of the relapsing fever episodes; further molecular testing was based on a multiplex dPCR assay routinely implemented in the IPRL-NCSU. The DNA sequencing of the ITS1 region supported infection with a *Babesia* species most similar to *B odocoilei* but infection with the closely related *B venatorum* could not be excluded, because GenBank currently has no ITS1 sequence for this species, known to infect ruminants and humans in Europe.^16^ To our knowledge, only 3 cases of babesiosis in dogs, caused by *B odocoilei*-like parasites in splenectomized dogs, have been reported in Japan.^24^ A novel strain, closely related to *B odocoilei*, also has been identified in splenectomized humans in Italy and Austria, and in *Ixodes ricinus* ticks in Slovenia.^11^ Although the temporal association of *Babesia* spp. amplification with the febrile episodes in this dog suggests a causal association, the role of *B odocoilei*-like infection remains unclear and additional studies must clarify its clinical relevance and that of *Babesia/Bartonella* coinfection. Importantly, anemia and thrombocytopenia were never documented in this dog during the 10-month study period. Despite negative blood qPCR for *Babesia* spp., the dog was repeatedly positive for *Piroplasma* 18S dPCR in blood or enrichment blood culture samples. The higher diagnostic sensitivity of dPCR has enabled detection of intraerythrocytic, stealth, *Babesia* pathogens causing persistent intravascular infections in animals and humans.^13^^,^^15^ Diagnosis of acute babesiosis in dogs has relied on blood smear cytology and serology, both of which were unrewarding in this dog. Cytology is compromised by the low circulating parasite level, particularly relevant with stealth *Babesia* pathogens, and serology cannot confirm active infection or differentiate between *Babesia* species because of cross-reactivity.^11^^,^^17^

Specific treatment for *Babesia* infection was not administered to this dog. Only one febrile episode occurred during the triple antibiotic regimen, with no relapses reported in the 20 months after treatment cessation. Azithromycin and doxycycline, which have antibabesial efficacy, in conjunction with an effective immune system response, may have eradicated infection.^11^ In babesiosis in humans, recovery may occur without medical treatment.^15^ Interestingly, serum ALP activity, which had been consistently increased, normalized in conjunction with antibiotic administration.

Both cats were tested to assess their potential role in the dog’s illness. The original household cat was once dPCR-positive for *Bartonella* spp., suggesting a possible infection source for the dog, but sequencing was not possible to confirm the species. Although the adopted feral cat was seroreactive to multiple *Bartonella* spp., no treatment was administered, according to recommendations for asymptomatic cats.^2^ The zoonotic potential of *Bartonella* spp. was carefully discussed with the owner. Although transmission of *Bartonella s*pecies to humans typically is associated with arthropod vectors, infected animals may represent a source of zoonotic exposure. Therefore, raising awareness is essential, including guidance on ectoparasite control and strategies to minimize the risk of transmission.

This case highlights the medical and microbiologic complexity of vector-borne coinfections in dogs and the potential limitations of conventional and advanced diagnostic testing modalities. It also provides the first clinical evidence of infection with a *Babesia* spp. that is phylogenetically closely related to *B odocoilei* in a dog in Greece. Broader recognition of zoonotic tick and other vector-borne pathogens in veterinary patients is crucial for optimal patient outcomes.

## Supplementary Material

Supplementary_Material_aalag174

## Off-label antimicrobial declaration

The authors declare no off-label use of antimicrobials.

## Institutional animal care and use committee or other approval declaration

The authors declare no institutional animal care and use committee or other approval was needed.

## Abbreviations

ALP: alkaline phosphatase
*Bvb*: *Bartonella vinsonii* subsp. *berkhoffii*
CAC-AUTh: Clinic of Companion Animal Medicine, Aristotle University of Thessaloniki
dPCR: digital polymerase chain reaction
IFA: indirect immunofluorescence assay
ITS: intergenic transcribed spacer
FNA: fine-needle aspirate
qPCR: quantitative real-time polymerase chain reaction
IPRL-NCSU: Intracellular Pathogens Research Laboratory, College of Veterinary Medicine, North Carolina State University
