# Supplementary material for: Bartonella vinsonii subsp. berkhoffii genotype III in a dog with recurrent fever and panniculitis coinfected with Babesia odocoilei-like sp
Source: J Vet Intern Med. 2026 Aug 24;40(4):aalag174. doi: 10.1093/jvimsj/aalag174 (PMC13502404; doi:10.1093/jvimsj/aalag174)
Supplement: Supplementary file 1 [file supplementary_material_aalag174.docx]

1. **Results of complete blood count (CBC) and serum biochemistry on 29 September 2023 – 1^st^ febrile episode.**

| CBC Parameter | Result | Reference Range |
| --- | --- | --- |
| Hematocrit | 57.58% | 37-55% |
| White blood cells | 4.89 x 10^9^/l | 5-17 x 10^9^/l |
| Neutrophils | 3.78 x 10^9^/l | 3-12 x 10^9^/l |
| Lymphocytes | 0.96 x 10^9^/l | 1-4.5 x 10^9^/l |
| Monocytes | 0.13 x 10^9^/l | 0.2-1.5 x 10^9^/l |
| Eosinophils | 0.02 x 10^9^/l | 0-0.9 x 10^9^/l |
| Basophils | 0.00 x 10^9^/l | 0-0.4 x 10^9^/l |
| Platelets | 229 x 10^9^/l | 165-500 x 10^9^/l |

| Biochemical Parameter | Result | Reference Range |
| --- | --- | --- |
| Total proteins | 7.2 g/dL | 5.4-8.2 g/dL |
| Blood urea nitrogen | 10 mg/dL | 7-25 mg/dL |
| Creatinine | 0.7 mg/dL | 0.3-1.4 mg/dL |
| Glucose | 103 mg/dL | 50-110 mg/dL |
| Alkaline phosphatase | 479 U/L | 200-150 U/L |
| Alanine aminotsansferase | 40 U/L | 10-118 U/L |

2. **Results of CBC, serum biochemistry, urine protein-to-creatinine ratio (upc), serology, cytology of various tissues, and cultures (blood, urine & synovial fluid) on 6 November 2023 – 2^nd^ febrile episode.**

| CBC Parameter | Result | Reference Range |
| --- | --- | --- |
| Hematocrit | 44.9% | 37-55% |
| White blood cells | 10.3 x 10^9^/l | 6-17 x 10^9^/l |
| Neutrophils | 6.8 x 10^9^/l | 3.9-8 x 10^9^/l |
| Lymphocytes | 1.8 x 10^9^/l | 1.3-4.1 x 10^9^/l |
| Monocytes | 1.1 x 10^9^/l | 0.2-1.1 x 10^9^/l |
| Eosinophils | 0.08 x 10^9^/l | 0-0.6 x 10^9^/l |
| Basophils | 0.02 x 10^9^/l | 0-0.1 x 10^9^/l |
| Platelets | 200 x 10^9^/l | 200-500 x 10^9^/l |

| Biochemical Parameter | Result | Reference Range |
| --- | --- | --- |
| Alkaline phosphatase | 70 U/L | 10-70 U/L |
| Alanine aminotsansferase | 36 U/L | 15-75 U/L |
| γ-GT | 2 U/L | 2-9 U/L |
| Total bilirubin | 0.2 mg/dL | 0.1-0.4 mg/dL |
| Albumin | 2.6 g/dL | 2.2-3.8 g/dL |

- Blood smear: normal findings
- Buffy coat examination: normal findings
- UPC: 0.09
- Snap 4Dx Plus Test (IDEXX): negative
- IFA testing for *Leishmania infantum*: negative
- Skin nodule FNA cytology: presence of red blood cells
- Mesenteric and medial iliac lymph nodes FNA cytology: mild pyogranulomatous lymphadenitis
- Spleen FNA cytology: normal findings
- Synovial fluid cytology: normal findings
- Blood, urine & synovial fluid cultures: negative

3. **Results of CBC, serum biochemistry, synovial fluid analysis and blood culture, blood qPCR for *Babesia* spp. and *Bartonella* spp. (IDEXX Laboratories; Germany) on 6 December 2023 – 3^rd^ febrile episode.**

| CBC Parameter | Result | Reference Range |
| --- | --- | --- |
| Hematocrit | 52.9% | 37-55% |
| White blood cells | 3.8 x 10^9^/l | 6-17 x 10^9^/l |
| Neutrophils | 1.1 x 10^9^/l | 3.9-8 x 10^9^/l |
| Lymphocytes | 1.2 x 10^9^/l | 1.3-4.1 x 10^9^/l |
| Monocytes | 0.1 x 10^9^/l | 0.2-1.1 x 10^9^/l |
| Eosinophils | 0.24 x 10^9^/l | 0-0.6 x 10^9^/l |
| Basophils | 0.02 x 10^9^/l | 0-0.1 x 10^9^/l |
| Platelets | 258 x 10^9^/l | 200-500 x 10^9^/l |

| Biochemical Parameter | Result | Reference Range |
| --- | --- | --- |
| Total proteins | 6.7 g/dL | 5.5-8 g/dL |
| Albumin | 3.6 g/dL | 2.9-4 g/dL |
| Blood urea nitrogen | 10 mg/dL | 10-38 mg/dL |
| Creatinine | 0.7 mg/dL | 0.7-1.3 mg/dL |
| Glucose | 98 mg/dL | 65-118 mg/dL |
| Total bilirubin | 0.2 mg/dL | 0.2-0.6 mg/dL |
| Alkaline phosphate | 712 U/L | 32-149 U/L |
| Alanine aminotsansferase | 49 U/L | 18-62 U/L |
| γ-GT | 2 U/L | 1-3 U/L |
| Phosphate (Phos) | 2.2 mg/dL | 2.2-6 mg/dL |
| Calcium (Ca) | 9 mg/dL | 8.6-10.9 mg/dL |
| Potassium (K) | 4 mEq/L | 3.7-5.9 mEq/L |
| Sodium (Na) | 142 mEq/L | 144-158 mEq/L |

- Blood smear: confirmation of neutropenia
- Synovial fluid cytology: normal findings
- Blood & synovial fluid cultures: negative
- qPCR for *Babesia* spp. (whole blood): negative (IDEXX Laboratories; Germany)
- qPCR for *Bartonella* spp. (whole blood): negative (IDEXX Laboratories; Germany)

4. **Results of subcutaneous FNA cytology on 12 January 2024 – 4^th^ febrile episode**

Sterile pyogranulomatous inflammation, consistent with a diagnosis of panniculitis.

5. **Results of CBC, serum biochemistry, serology for *B. henselae,* histopathology and Warthin-Starry silver staining, qPCR for *Bartonella* spp. of the excised right thoracic wall nodule on 22 January 2024 – 5^th^ febrile episode**

| CBC Parameter | Result | Reference Range |
| --- | --- | --- |
| Hematocrit | 44.5% | 37-55% |
| White blood cells | 14.7 x 10^9^/l | 6-17 x 10^9^/l |
| Neutrophils | 7.6 x 10^9^/l | 3.9-8 x 10^9^/l |
| Lymphocytes | 3.2 x 10^9^/l | 1.3-4.1 x 10^9^/l |
| Monocytes | 2.3 x 10^9^/l | 0.2-1.1 x 10^9^/l |
| Eosinophils | 0.28 x 10^9^/l | 0-0.6 x 10^9^/l |
| Basophils | 0.04 x 10^9^/l | 0-0.1 x 10^9^/l |
| Platelets | 308 x 10^9^/l | 200-500 x 10^9^/l |

| Biochemical Parameter | Result | Reference Range |
| --- | --- | --- |
| Total protein | 6.1 g/dL | 5.5-8 g/dL |
| Albumin | 3.3 g/dL | 2.9-4 g/dL |
| Blood urea nitrogen | 14 mg/dL | 10-38 mg/dL |
| Creatinine | 0.6 mg/dL | 0.7-1.3 mg/dL |
| Alkaline phosphate | 671 U/L | 32-149 U/L |
| Alanine aminotsansferase | 22 U/L | 18-62 U/L |

- Blood smear: normal findings
- *Bartonella henselae* Antibodies (IFAT): 1:40 (Reference value: <1:20)
- Histopathology of the excised thoracic wall nodule: pyogranulomatous inflammation of adipose tissue without extension to the overlying dermis, consistent with panniculitis. The infiltrate was predominantly neutrophilic, with a smaller population of macrophages and occasional multinucleated giant cells, with accompanying edema, chronic hemorrhage, and reactive fibroplasia and fibrosis (Figure 3). Warthin-Starry silver staining failed to reveal organisms. Additional histochemical stains were performed, including PAS, which did not reveal fungi, and Ziehl–Neelsen, which did not reveal mycobacteria
- qPCR for *Bartonella* spp. of the excised right thoracic wall nodule: positive

6. **Results of CBC, serum biochemistry, on 10 February 2024 – 6^th^ febrile episode**

| CBC Parameter | Result | Reference Range |
| --- | --- | --- |
| Hematocrit | 47.9% | 37-55% |
| White blood cells | 6.7 x 10^9^/l | 6-17 x 10^9^/l |
| Neutrophils | 3.9 x 10^9^/l | 3.9-8 x 10^9^/l |
| Lymphocytes | 2.1 x 10^9^/l | 1.3-4.1 x 10^9^/l |
| Monocytes | 0.3 x 10^9^/l | 0.2-1.1 x 10^9^/l |
| Eosinophils | 0.3 x 10^9^/l | 0-0.6 x 10^9^/l |
| Basophils | 0.01 x 10^9^/l | 0-0.1 x 10^9^/l |
| Platelets | 210 x 10^9^/l | 200-500 x 10^9^/l |

| Biochemical Parameter | Result | Reference Range |
| --- | --- | --- |
| Alkaline phosphate | 529 U/L | 32-149 U/L |
